# Exploring risk factors of short-term readmission in heart failure patients: A cohort study

**DOI:** 10.3389/fendo.2022.1024759

**Published:** 2022-11-28

**Authors:** Xiao Na Niu, He Wen, Nan Sun, Ran Zhao, Ting Wang, Yan Li

**Affiliations:** Department of Cardiology, Tangdu Hospital, The Second Affiliated Hospital of Air Force Medical University, Xi’an, China

**Keywords:** heart failure (HF), prognosis, risk factors, readmission, a cohort study

## Abstract

**Background:**

The risk of all-cause mortality in patients with heart failure (HF) has been studied previously. Readmission risk of HF patients was rarely explored. Thus, we aimed to explore early warning factors that may influence short-term readmission of HF patients.

**Methods:**

The data of this study came from an HF database in China. It was a retrospective single-center observational study that collected characteristic data on Chinese HF patients by integrating electronic medical records and follow-up outcome data. Eventually, 1,727 patients with HF were finally included in our study.

**Results:**

In our study, the proportion of HF patients with New York Heart Association (NYHA) class II, III, and IV HF were 17.20%, 52.69%, and 30.11%, respectively. The proportion of patients with readmission within 6 months and readmission within 3 months was 38.33% and 24.20%, respectively. Multivariate logistic regression showed that NYHA class (*p*_III_ = 0.028, *p*_IV_ < 0.001), diabetes (*p* = 0.002), Cr (*p* = 0.003), and RDW-SD (*p* = 0.039) were risk factors for readmission within 6 months of HF patients. NYHA class (*p*_III_ = 0.038, *p*_IV_ < 0.001), CCI (*p* = 0.033), Cr (*p* = 0.012), UA (*p* = 0.042), and Na (*p* = 0.026) were risk factors for readmission within 3 months of HF patients.

**Conclusions:**

Our study implied risk factors of short-term readmission risk in patients with HF, which may provide policy guidance for the prognosis of patients with HF.

## Introduction

Heart failure (HF) refers to a syndrome in which cardiac output is reduced due to primary heart damage and cannot meet the needs of tissue metabolism under the condition of normal venous return. HF is the end stage of heart disease caused by various etiologies, which is a serious stage in which the heart loses its ability to compensate due to disease, overwork, and so on (1, 2). HF can be divided into heart failure with reduced ejection fraction (HFrEF), heart failure with preserved ejection fraction (HFpEF), and heart failure in the middle range of ejection fraction (HFmrEF) according to European Society of Cardiology (ESC) 2016 HF guidelines (3). HF is mostly caused by heart diseases with high incidence, such as coronary heart disease, rheumatic heart disease, myocarditis, and diabetic cardiomyopathy. Some diseases that can increase the burden on the sick heart can induce HF, such as pregnancy, fatigue, and rapid intravenous rehydration (4, 5), which are the inducing factors of acute HF. HF is a rapidly growing cardiovascular disease affecting more than 37.7 million people worldwide, with a significant increase in the number of patients with HF (6–8).

In high economic countries like the USA, HF has affected over 6 million adult populations (≥20 years) (9). According to the 2013 mortality report, more than 2,200 patients die of cardiovascular diseases every day. HF is the root cause of most deaths (10). HF-related mortality is currently higher in low- and middle-income countries than in high-income countries (11). Therefore, we should strengthen the research on patients with HF and pay attention to the prognosis of patients with HF.

Previous studies have developed prognostic models to assess the risk of all-cause mortality in patients with chronic HF (12, 13). Most investigators use survival and death in patients with HF as prognostic outcomes. However, few investigators used readmission as an outcome measure; traditional approach does not take into account the burden of recurrence (14). Currently, there are few studies on the risk factors for readmission in patients with HF. HF patients are also frequently readmitted, with a 60- to 90-day readmission rate of 29%, and congestive HF patients are frequently readmitted to the hospital because of worsening symptoms, with a reported 3- to 6-month readmission rate as high as 30% to 50% (15, 16). The increasing number of readmission is recognized as an important marker of quality of care and highlights the many vulnerabilities of patients as they transition from the hospital to the community. Reducing readmission has the potential not only to improve patient prognosis, but also to reduce costs (17). Many hospitalizations are considered preventable, and identifying those patients most at risk and developing interventions to prevent readmission has been a constant focus of clinicians and policymakers. The severity of HF can affect the overall prognosis of patients with HF, resulting in significant functional impairment and symptom burden (10, 11). Contributing factors to poor outcomes in HF include the presence of comorbidities, disease severity, and inadequate health literacy. Studies have also shown that patients with HF have poor adherence to self-care advice and poor outcomes (18, 19). Therefore, we should pay attention to the readmission of patients with HF. In our study, the HF patients in the database were selected as the research objects to explore the influencing factors of short-term readmission of HF patients, and to provide some suggestions for the prognosis of HF patients.

## Materials and methods

### Study design and population

A total of 1,727 subjects were included in this study. The participants in our study were from a Chinese HF database. The research objects included in the database were from Zigong Hospital in Sichuan Province from December 2016 to June 2019. It was a cohort study to collect information about the characteristics of HF patients in China by integrating electronic medical records and follow-up outcome data. This study was approved by the Ethics Committee of the Fourth People’s Hospital of Zigong (approval number: 2020-010). Due to the retrospective design of this study, informed consent was waived. The study complies with the Declaration of Helsinki. The database is available at PhysioNet (https://doi.org/10.13026/8a9e-w734) (20, 21).

Independent variables of our study were broadly divided into three categories: general demographic data (age, BMI, and gender), basic illness data (myocardial infarction, congestive HF, peripheral vascular disease, and so on), and laboratory test indicators (Cr, UA, GFR, and so on). The Charlson comorbidity index (CCI) was calculated by summing all comorbidity points in the database (22). Comorbidities include myocardial infarction, congestive HF, peripheral vascular disease, cerebrovascular disease, dementia, COPD, connective tissue disease, peptic ulcer disease, diabetes, moderate to severe chronic kidney disease, hemiplegia, leukemia, malignant lymphoma, solid tumors, liver disease, and AIDS (22).

### Statistical analysis

Categorical variables were expressed as number and percentage. Continuous variables were described using median and interquartile range (IQR) for non-normally distributed data. Non-parametric test for continuous variables and the *χ* ^2^ test for categorical variables were used to compare differences between the shorter-term readmission group and the non-readmission group. Univariate logistic regression was used to explore risk factors that may influence short-term readmission in patients with HF. Afterwards, multivariate logistic regression was used to further screen out the predictors of readmission in patients with HF. Finally, the violin plot was used to simulate the distribution of the predictor factors, and the restricted cubic spline (RCS) was used to simulate the nonlinear relationship between the predictor factors and short-term readmission risk in patients with HF. Univariate analysis and multivariate analysis were performed in SPSS 23.0 software package (SPSS, Chicago, Illinois, USA), forest plot and RCS were performed in R version 4.1.1 (R Foundation for Statistical Computing, Austria), and violin diagram was performed in GraphPad Prism, version 8.0 (GraphPad Software, San Diego, CA, USA). A *p*-value < 0.05 (two-sided) was considered statistically significant.

## Results

The proportion of male participants was 41.52% and the proportion of participants ≤ 59 years old was 8.92%. In readmission within the 3-month group, the proportion of male participants was 41.87% and the proportion of participants ≤ 59 years was 6.94%. In readmission within the 6-month group, the proportion of male participants was 42.75% and the proportion of participants ≤ 59 years was 7.55%. There were no significant differences in gender and age composition ratio between the 3-month readmission group and the non-3-month readmission group (*p*_gender_ = 0.868; *p*_age_ = 0.103). There were also no significant differences in the gender and age composition ratio between the 6-month readmission group and the non-6-month readmission group (*p*_gender_ = 0.413; *p*_age_ = 0.117). In readmission within the 6-month group, NYHA class II, III, and IV patients with HF were 89, 341, and 232. In readmission within the 3-month group, the proportions of NYHA class II, III, and IV HF patients were 11.96%, 50.72%, and 37.32%, respectively. There were statistically significant differences in the NYHA class composition ratio between the 3/6-month readmission group and the non-3/6-month readmission group (*p* < 0.001). In readmission within the 3-month group, the proportion of participants with myocardial infarction, congestive HF, peripheral vascular disease, and COPD were 7.18%, 91.63%, 5.98%, and 13.88%, respectively. There were no significant differences in the composition ratio of patients with myocardial infarction, congestive HF, peripheral vascular disease, or COPD between the 3-month readmission group and the non-3-month readmission group (*p*_myocardial infarction_ = 0.874; *p*_congestive heart failure_ = 0.230; *p*_peripheral vascular disease_ = 0.280; *p*_COPD_ = 0.122). In readmission within the 6-month group, there were 47, 614, 36, and 81 participants with myocardial infarction, congestive HF, peripheral vascular disease, and COPD, respectively. The composition ratios of patients with myocardial infarction, congestive HF, peripheral vascular disease, or COPD in the two groups of patients with readmission within 6 months were consistent with that within 3 months (*p*-values were all greater than 0.05). Of the total participants, 401 research subjects had diabetes; in the 3-month readmission group, 28.23% HF patients had diabetes; in the 6-month readmission group, the proportion of participants with diabetes was 27.79%. The proportion of HF patients with a CCI score greater than 2 points in the short-term readmission group was higher than that in the non-short-term readmission group (*p* < 0.001) (**Table 1**).

**Table 1:** Univariate analysis of categorical variables.

Variables	Total (*N* = 1,727)	No readmission within 6 months (*N* = 1,065)	Readmission within 6 months (*N* = 662)	*p*-value	No readmission within 3 months (*N* = 1,309)	Readmission within 3 months (*N* = 418)	*p*-value
Age (years)				0.117			0.103
≤59	154 (8.92%)	104 (9.77%)	50 (7.55%)		125 (9.55%)	29 (6.94%)	
>59	1,573 (91.08%)	961 (90.23%)	612 (92.45%)		1,184 (90.45%)	389 (93.06%)	
Gender				0.413			0.868
Male	717 (41.52%)	434 (40.75%)	283 (42.75%)		542 (41.41%)	175 (41.87%)	
Female	1,010 (58.48%)	631 (59.25%)	379 (57.25%)		767 (58.59%)	243 (58.13%)	
NYHA class				<0.001			<0.001
II	297 (17.20%)	208 (19.53%)	89 (13.44%)		247 (18.87%)	50 (11.96%)	
III	910 (52.69%)	569 (53.43%)	341 (51.51%)		698 (53.32%)	212 (50.72%)	
IV	520 (30.11%)	288 (27.04%)	232 (35.05%)		364 (27.81%)	156 (37.32%)	
Myocardial infarction				0.750			0.874
No	1,600 (92.65%)	985 (92.49%)	615 (92.90%)		1,212 (92.59%)	388 (92.82%)	
Yes	127 (7.35%)	80 (7.51%)	47 (7.10%)		97 (7.41%)	30 (7.18%)	
Congestive heart failure				0.812			0.230
No	122 (7.06%)	74 (6.95%)	48 (7.25%)		87 (6.65%)	35 (8.37%)	
Yes	1,605 (92.94%)	991 (93.05%)	614 (92.75%)		1,222 (93.35%)	383 (91.63%)	
Peripheral vascular disease				0.490			0.280
No	1,641 (95.02%)	1,015 (95.31%)	626 (94.56%)		1,248 (95.34%)	393 (94.02%)	
Yes	86 (4.98%)	50 (4.69%)	36 (5.44%)		61 (4.66%)	25 (5.98%)	
COPD				0.624			0.122
No	1,524 (88.25%)	943 (88.54%)	581 (87.76%)		1,164 (88.92%)	360 (86.12%)	
Yes	203 (11.75%)	122 (11.46%)	81 (12.24%)		145 (11.08%)	58 (13.88%)	
CCI score				<0.001			<0.001
0	1,322 (76.55%)	847 (79.53%)	475 (71.75%)		1,029 (78.61%)	293 (70.10%)	
1	405 (23.45%)	218 (20.47%)	187 (28.25%)		280 (21.39%)	125 (29.90%)	
Diabetes				<0.001			0.005
No	1,326 (76.78%)	848 (79.62%)	478 (72.21%)		1,026 (78.38%)	300 (71.77%)	
Yes	401 (23.22%)	217 (20.38%)	184 (27.79%)		283 (21.62%)	118 (28.23%)	

COPD, Chronic obstructive pulmonary disease; CCI, Charlson comorbidity index, 0: ≤2 points; 1: >2 points.

The BMI of readmission and non-readmission patients within 6 months were 20.40 kg/m^2^ and 20.89 kg/m^2^. There was no statistically significant difference in BMI between the two groups (*p* = 0.073). However, the difference in BMI between the 3-month readmission group and the non-3-month readmission group was statistically significant (*p* = 0.036). The levels of renal function indexes Cr, Urea, UA, and GFR of all selected HF patients were 87.10 μmol/L, 8.01 mmol/L, 454.00 μmol/L, and 64.93 ml/min/1.73 m^2^, respectively. There were significant differences in Cr, Urea, UA, and GFR between the short-term readmission group and the non-readmission group (*p*_Cr_ < 0.001; *p*_Urea_ < 0.001; *p*_UA_ < 0.001; *p*_GFR_ < 0.001). In readmission within the 3-month group, the median value of RDW-CV was 14.60%; the median value of RDW-SD was 48.40 fl. In readmission within the 6-month group, the median value of RDW-CV was 14.40%; the median value of RDW-SD was 48.10 fl. There were statistically significant differences in RDW-CV and RDW-SD levels between the short-term readmission group and the non-readmission group. In the 3-month readmission group, the average level of HGB in the HF patients was 115.00 g/L; in the 6-month readmission group, the average level of HGB in the HF patients was 116.00 g/L. There were statistically significant differences in HGB levels between the short-term readmission group and the non-readmission group (*p*_readmission within 3 months_ = 0.009; *p*_readmission within 6 months_ = 0.007). In the 3-month readmission group, the levels of Ca, K, and Na in patients with HF were 2.29 mmol/L, 3.92 mmol/L, and 138.35 mmol/L. There were statistically significant differences in K and Na levels between the readmission within the 3-month group and the non-readmission within the 3-month group (*p*_K_ = 0.003; *p*_Na_ < 0.001). There were statistically significant differences in Ca, K, and Na levels between the readmission within the 6-month group and the non-readmission within the 6-month group (*p*_Ca_ = 0.041; *p*_K_ = 0.009; *p*_Na_ = 0.007). There were significant differences in the levels of cholesterol and LDL-C between the short-term readmission group and the non-short-term readmission group, and there were no statistical differences in the levels of TG and HDL-C between the short-term readmission group and the non-short-term readmission group (**Table 2**).

**Table 2:** Univariate analysis of continuous variables.

Variables	Total (*N* = 1,727)	No readmission within 6 months (*N* = 1,065)	Readmission within 6 months (*N* = 662)	*p*-value	No readmission within 3 months (*N* = 1,309)	Readmission within 3 months (*N* = 418)	*p*-value
BMI (kg/m^2^)	20.78 (18.49, 23.44)	20.89 (18.66, 23.46)	20.40 (18.37, 23.44)	0.073	20.82 (18.61, 23.56)	20.26 (18.34, 22.89)	0.036
Cr (μmol/L)	87.10 (64.70, 122.60)	83.10 (63.05, 115.60)	92.90 (69.08, 130.93)	<0.001	84.60 (63.75, 116.65)	98.90 (70.48, 135.90)	<0.001
Urea (mmol/L)	8.01 (5.86, 11.46)	7.57 (5.67, 10.86)	8.61 (6.24, 12.31)	<0.001	7.62 (5.75, 10.98)	9.13 (6.41, 13.20)	<0.001
UA (μmol/L)	454.00 (359.00, 567.00)	444.00 (352.00, 557.00)	474.00 (374.00, 600.50)	<0.001	442.00 (354.50, 556.50)	486.50 (384.00, 627.00)	<0.001
GFR (mL/min/1.73 m^2^)	64.93 (41.67, 90.09)	68.84 (44.51, 93.62)	58.03 (39.20, 83.09)	<0.001	67.88 (43.94, 92.24)	55.96 (37.28, 81.04)	<0.001
RDW-CV (%)	14.40 (13.60, 15.60)	14.40 (13.50, 15.50)	14.40 (13.70, 15.80)	0.029	14.30 (13.60, 15.50)	14.60 (13.80, 15.90)	0.001
RDW-SD (fl)	47.80 (45.20, 51.40)	47.50 (44.90, 50.90)	48.10 (45.48, 52.10)	0.004	47.60 (45.00, 50.90)	48.40 (45.60, 52.80)	0.001
HGB (g/L)	117.00 (101.00, 131.00)	118.00 (102.00, 133.00)	116.00 (100.00, 129.00)	0.007	118.00 (102.00, 132.00)	115.00 (99.00, 128.00)	0.009
Ca (mmol/L)	2.29 (2.18, 2.40)	2.28 (2.17, 2.39)	2.30 (2.19, 2.41)	0.041	2.29 (2.18, 2.40)	2.29 (2.19, 2.39)	0.688
K (mmol/L)	3.87 (3.53, 4.30)	3.84 (3.49, 4.28)	3.90 (3.58, 4.37)	0.009	3.84 (3.50, 4.27)	3.92 (3.62, 4.41)	0.003
Na (mmol/L)	139.00 (136.00,141.40)	139.20 (136.40, 141.70)	138.80 (135.50, 141.00)	0.007	139.20 (136.40, 141.65)	138.35 (135.00, 141.00)	<0.001
TG (mmol/L)	0.95 (0.71, 1.29)	0.97 (0.73, 1.28)	0.91 (0.69, 1.31)	0.099	0.96 (0.71, 1.29)	0.92 (0.70, 1.33)	0.587
Cholesterol (mmol/L)	3.58 (2.96, 4.30)	3.63 (3.03, 4.37)	3.46 (2.87, 4.23)	0.004	3.62 (3.02, 4.33)	3.44 (2.83, 4.16)	0.004
LDL-C (mmol/L)	1.75 (1.32, 2.28)	1.78 (1.36, 2.33)	1.69 (1.26, 2.20)	0.006	1.77 (1.34, 2.31)	1.66 (1.25, 2.18)	0.010
HDL-C (mmol/L)	1.07 (0.86, 1.30)	1.08 (0.86, 1.31)	1.06 (0.86, 1.30)	0.546	1.08 (0.87, 1.32)	1.06 (0.84, 1.26)	0.093

BMI, body mass index; Cr, creatinine; UA, uric acid; GFR, glomerular filtration rate; RDW-CV, coefficient of variation of red blood cell distribution width; RDW-SD, standard deviation of red blood cell distribution width; HGB, hemoglobin; Ca, calcium; K, potassium; Na, sodium; TG, triglyceride; LDL-C, low-density lipoprotein cholesterol; HDL-C, high-density lipoprotein cholesterol.

**Figure 1:**
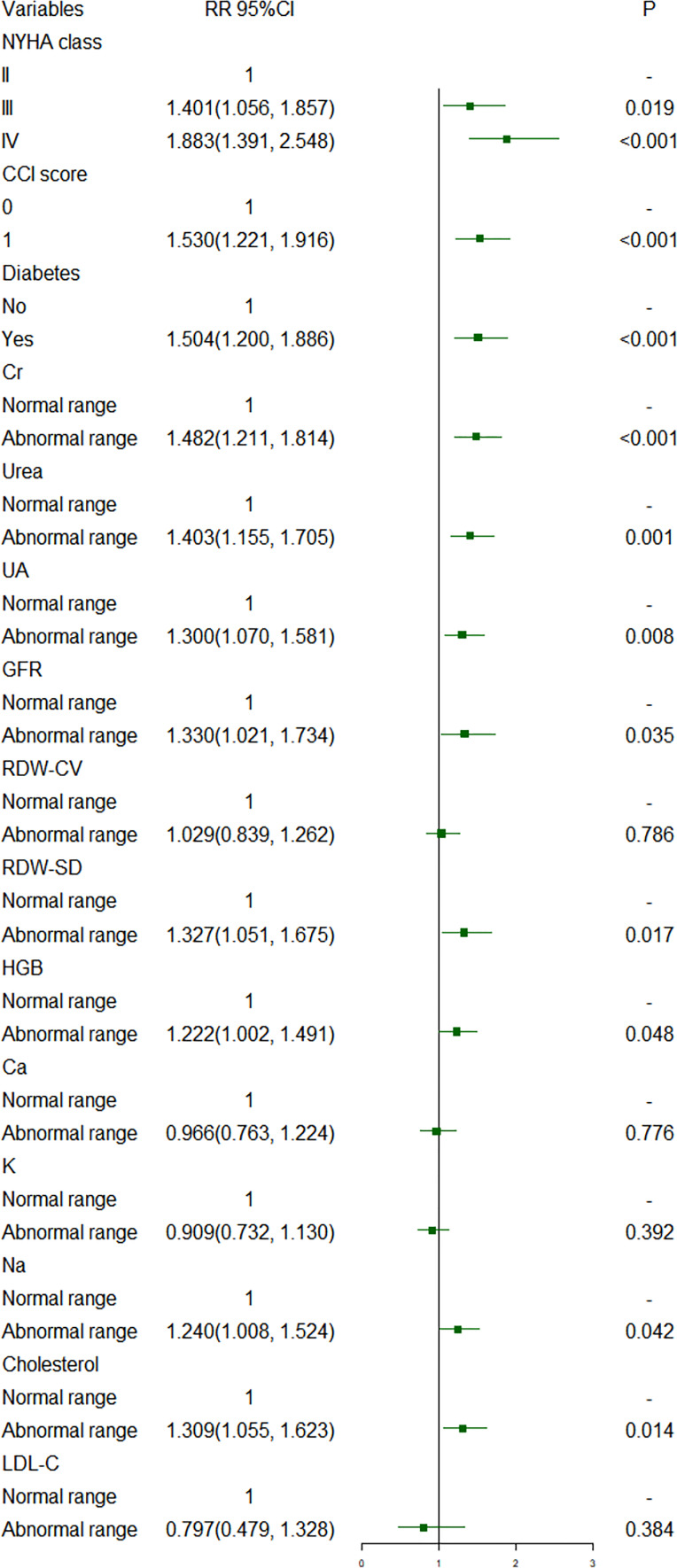
Risk factors for readmission within 6 months of heart failure patients: univariate logistic regression (Normal range: Cr: 44-110µmol/L; Urea: 1.7-8.3 mmol/L; UA: 150-440 µmol/L; GFR: 90-120 mL/min/1.73 m^^2^; RDw-CV: 0-15%; RDW-SD: 40-53 fL; HGB: 110-160g/L; Ca; 2.11-2.52 mmol/L; K: 3.5-5.3 mmol/L; Na: 137-147 mmol/L; Cholesterol: 2.90-5.68 mmol/L; LDL-C: 0-3.36 mmol/L).

**Figure 2:**
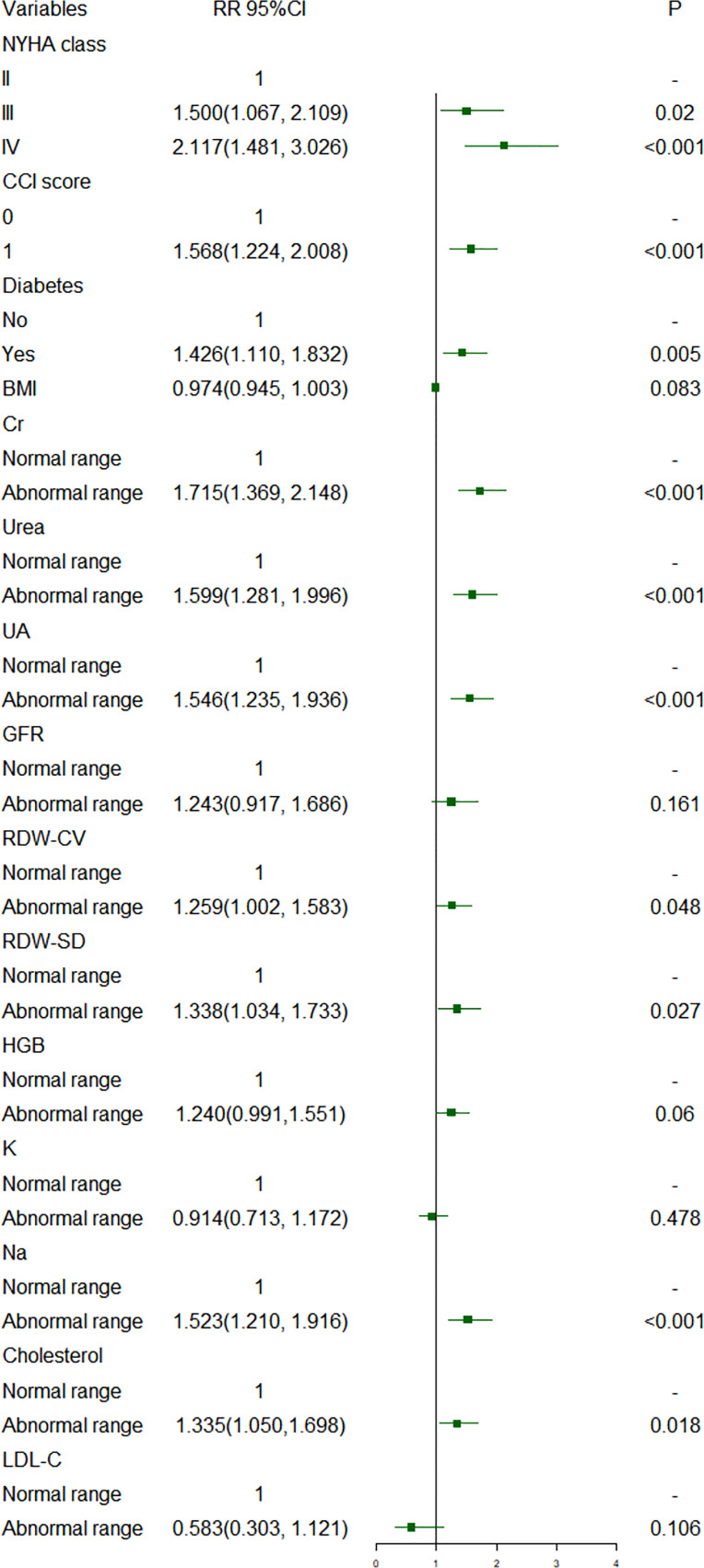
Risk factors for readmission within 3 months of heart failure patients: univariate logistic regression.

**Figure 3:**
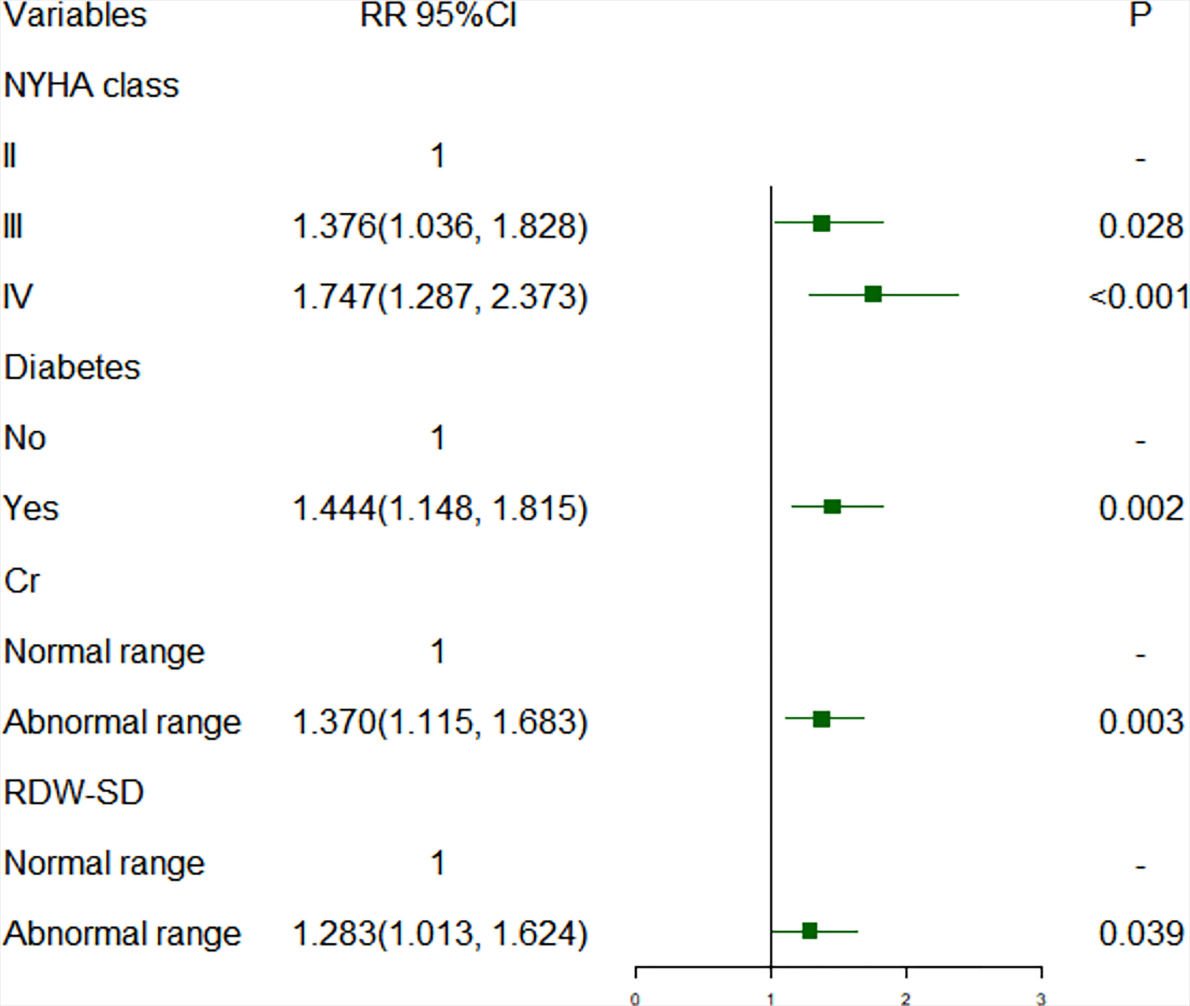
Risk factors for readmission within 6 months of heart failure patients: multivariate logistic regression.

**Figure 4:**
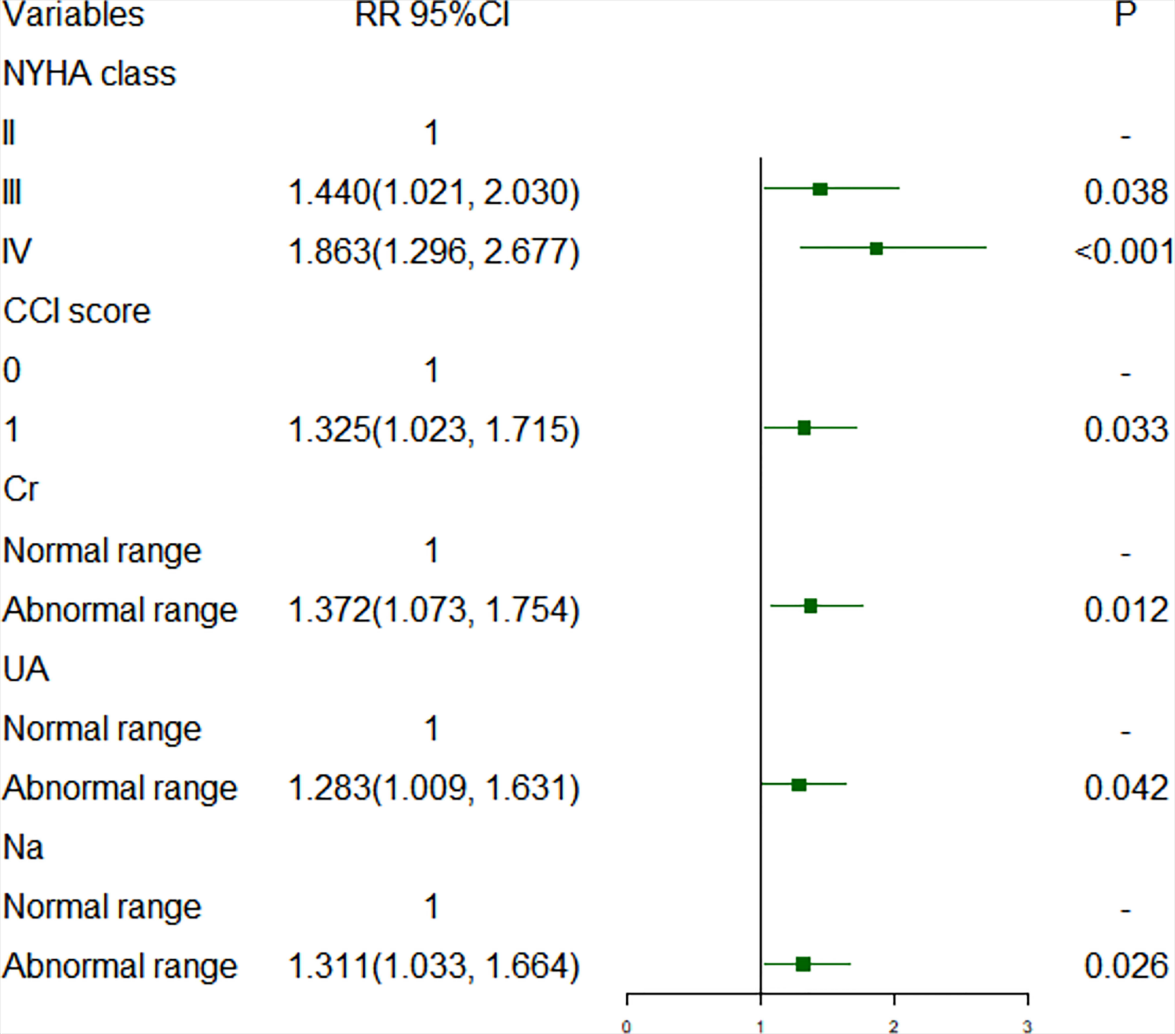
Risk factors for readmission within 3 months of heart failure patients: multivariate logistic regression.

**Figure 5:**
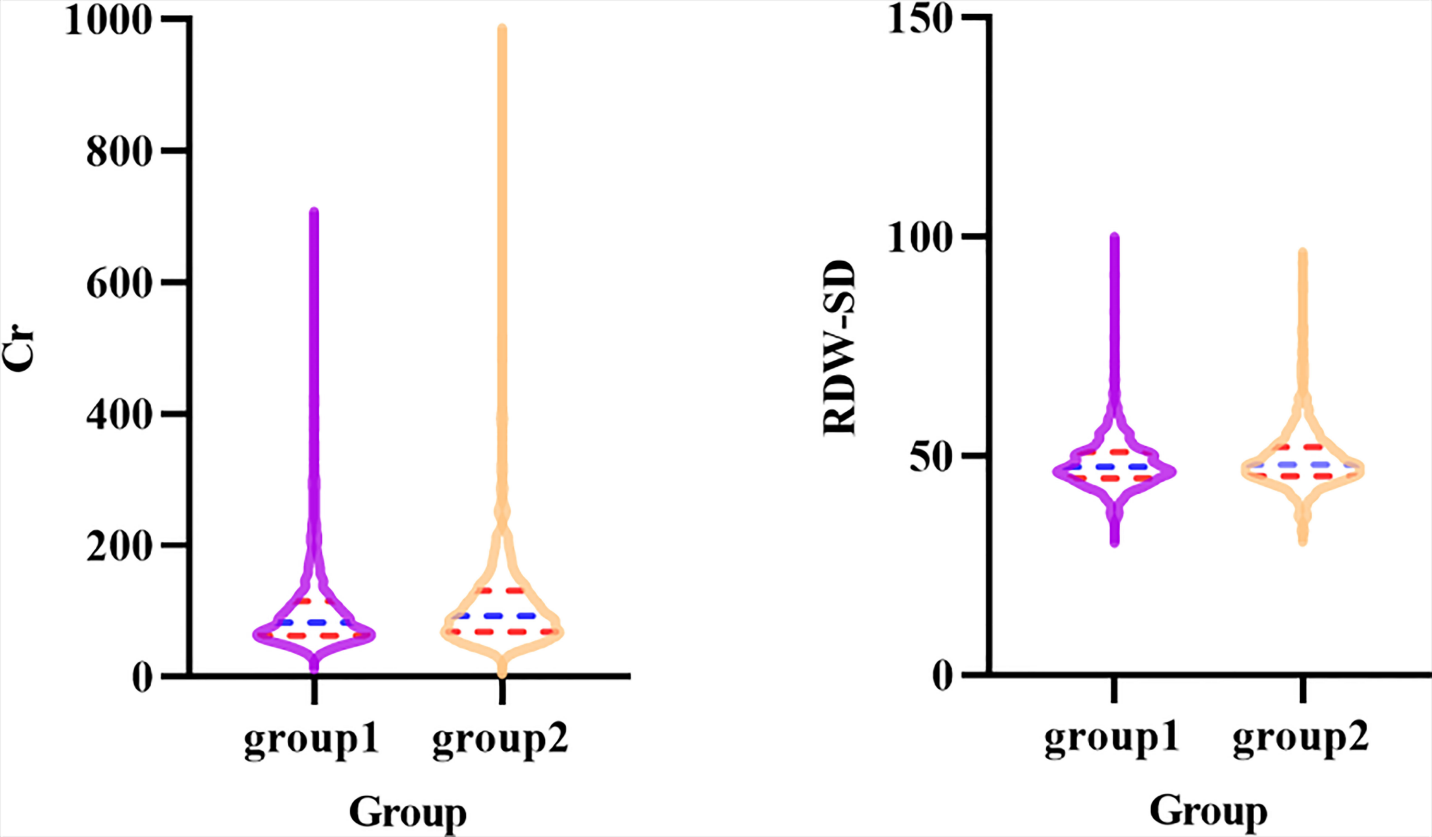
Violin plot of Cr and RDW-SD in patients with heart failure in readmission and non-readmission within 6 months groups (Group1: non-readmission; Group2: readmission).

**Figure 6:**
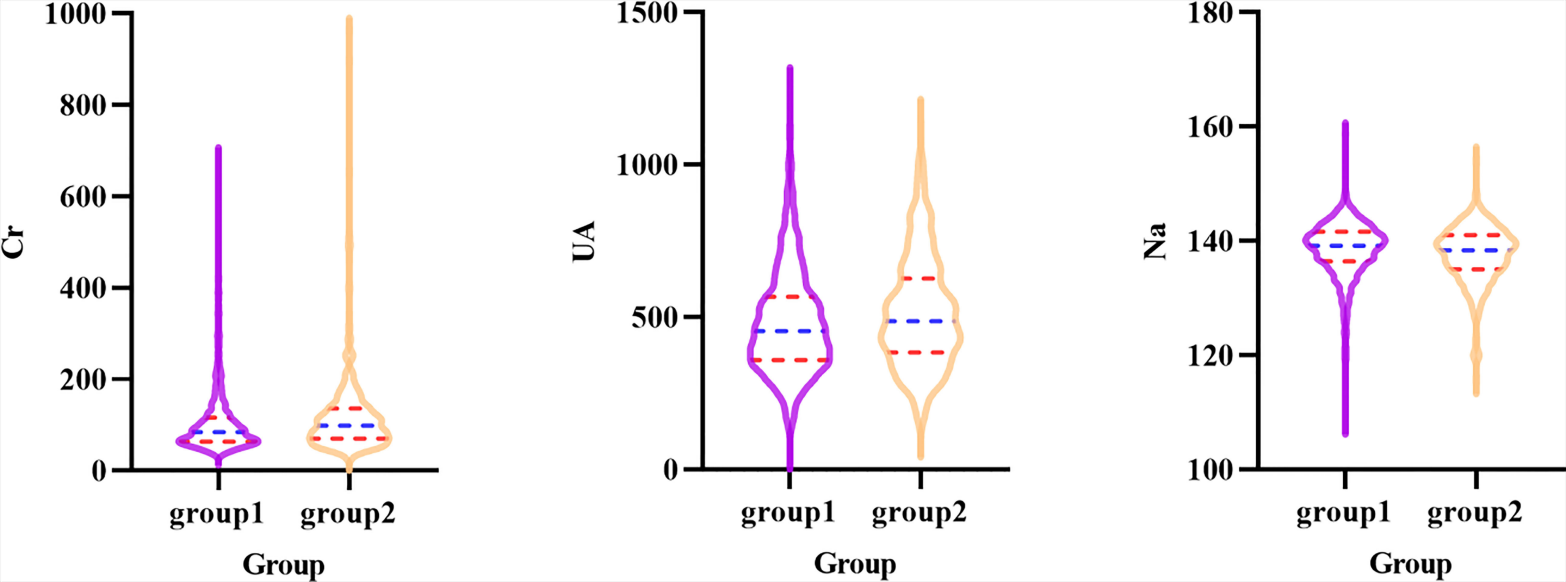
Violin plot of Cr, UA and Na in patients with heart failure in readmission and non-readmission within 3 months groups (Group1: non-readmission; Group2: readmission).

**Figure 7:**
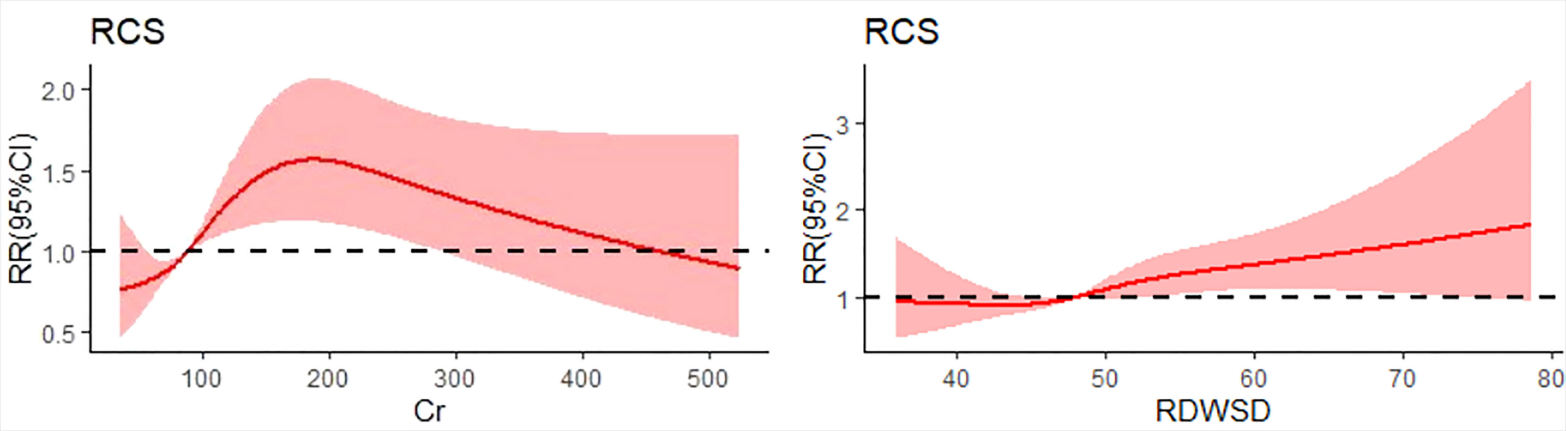
Restricted cubic spline plot of the relationship between readmission and influencing factors in heart failure patients within 6 months. (RDWSD: standard deviation of red blood cell distribution width).

**Figure 8:**
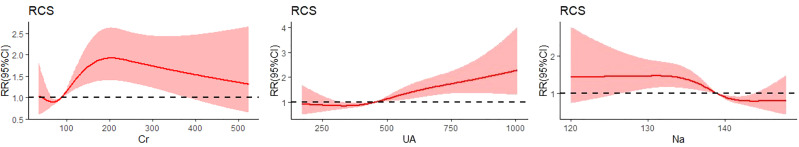
Restricted cubic spline plot of the relationship between readmission and influencing factors in heart failure patients within 3 months.

**Figures 1**, **2** show short-term prognostic risk factors in patients with HF. Within 6 months, the risk of readmission in HF patients with NYHA class III was 1.401 times that of HF patients with NYHA class II; the risk of readmission in HF patients with NYHA class IV was 1.883 times that of HF patients with NYHA class II; the risk of readmission in HF patients with diabetes was 1.504 times that of HF patients without diabetes. Within 3 months, the risk of readmission in HF patients with NYHA class III was 1.500 times that of HF patients with NYHA class II; the risk of readmission in HF patients with NYHA class IV was 2.117 times that of HF patients with NYHA class II; the risk of readmission in HF patients with diabetes was 1.426 times the risk of readmission in HF patients without diabetes. According to the provided critical range, the laboratory test indexes Cr, Urea, UA, GFR, RDW-CV, RDW-SD, HGB, Ca, K, Na, TG, cholesterol, and LDL-C were divided into two groups: the normal group and the abnormal group. Subsequently, significant factors with *p* < 0.05 from the univariate logistic regression were included in the multivariate analysis, using a forward, conditional approach to perform the multivariate logistic regression analysis. **Figures 3**, **4** show the results of multivariate logistic regression. Within 6 months, the risk of readmission in HF patients with NYHA class III was 1.376 times that of HF patients with NYHA class II; the risk of readmission in HF patients with NYHA class IV was 1.747 times that of HF patients with NYHA class II; the risk of readmission in HF patients with diabetes was 1.444 times that of HF patients without diabetes; the risk of readmission in HF patients with abnormal Cr was 1.370 times that of HF patients with normal Cr; the risk of readmission in HF with abnormal RDW-SD was 1.283 times that of HF patients with normal RDW-SD. Within 3 months, the risk of readmission in HF patients with NYHA class III was 1.440 times that of HF patients with NYHA class II; the risk of readmission in HF patients with NYHA class IV was 1.863 times that of HF patients with NYHA class II; the risk of readmission in HF patients with CCI > 2 points was 1.325 times that of HF patients with CCI ≤ 2 points; the risk of readmission in HF with abnormal UA was 1.283 times that of HF patients with normal UA; the risk of readmission in HF patients with abnormal Cr was 1.372 times that of HF patients with normal Cr; the risk of readmission in HF with abnormal Na was 1.311 times that of HF patients with normal Na. **Figures 5**, **6** show the distribution of risk factors for short-term prognosis in patients with HF. **Figures 7**, **8** show the nonlinear relationship between short-term prognosis and influencing factors.

## Discussion

HF is a clinical syndrome usually caused by structural and/or functional cardiac abnormalities, resulting in decreased cardiac output and/or increased intracardiac pressure (23). In 2021, the world’s leading scientific body proposed a consensus on a universal definition and classification of HF. HF has been defined as a global pandemic, with an estimated 64.3 million people worldwide suffering from HF in 2017 (24). HF affects more than 4 million people in China, with 500,000 new cases diagnosed each year (25, 26). With increases in coronary artery disease, hypertension, and an aging population, the incidence and prevalence of HF in China are expected to increase further, similar to many other low-income and middle-income countries (27–30). Among 22,158 participants recruited by the China Hypertension Survey (CHS), the prevalence of HF was 1.3% (31). The burden of HF in India is high, with an estimated population range between 1.3 million and 4.6 million (32). Data on the prevalence of HF in South Asia are very limited, and the estimated range is between 1.3% and 6.7% (33). It is estimated at 5% in Indonesia, 1%–2% in the Philippines, 0.6% in South Korea, and 0.4% in Thailand (34). The prevalence in Australia is estimated to be between 1% and 2% (35). Despite the growing importance of HF in China, representative information on these patients is still lacking. An important step is who is at high risk of readmission. The increase in readmission is a global concern, placing a considerable burden on patients, treatment costs, and hospital resources. The 30-day readmission rate for HF patients in the United States between 1993 and 2006 increased from 17% to 20%, and current readmission patterns tend to be related to length of stay and clinical factors, such as age and comorbidities (36). The government has experimented with public reporting and financial incentives aimed at reducing readmission rates. However, readmission rates for patients with HF were not significantly reduced.

Previous studies on the prognosis of patients with HF have mostly used all-cause mortality as an outcome variable, and few researchers have studied readmission of patients with HF in our country. Our research may be able to make up for the shortage of prognostic research on readmission of patients with HF. Data from a large clinical trial of patients with congestive HF confirmed the prognostic importance of several baseline characteristics reported in other studies (12, 37), which identified 25 independent predictors; the 12 most important factors—age, NYHA class, eGFR, LVEF, COPD, sex, SBP, diabetes, hemoglobin level, uricemia, aortic stenosis, and BMI—contained most of the prognostic information. Overall, increasing age was consistently associated with worse outcomes in several studies and was the strongest risk factor, followed by NYHA class. Most of the subjects in this study were older than 50 years old. Therefore, this study did not find a relationship between age and readmission risk. The third strongest predictor was eGFR. In fact, the association between poorer outcomes and lower eGFR was only evident with eGFR < 60 (13). A foreign study established a readmission risk model for patients with congestive HF, but there are few domestic studies on HF readmission (38). Therefore, this study decided to investigate the risk factors for readmission in patients with HF. In our study, we found that NYHA class, diabetes, CCI, Cr, RDW-SD, UA, and Na were associated with short-term readmission risk in patients with HF. NYHA class and diabetes had the greatest impact on patient readmission risk; Cr had a greater impact on the risk of short-term readmission. Patients with NYHA class III or IV, a CCI score greater than 2 points, and diabetes had a higher risk of short-term readmission. Cr, RDW-SD, UA, and Na showed a nonlinear relationship with short-term readmission risk. The risk of short-term readmission in HF patients with abnormal Cr, RDW-SD, UA, and Na was higher than that in patients with normal Cr, RDW-SD, UA, and Na. There are a few references to compare the results of two independent samples *t*-test and univariate logistic regression analysis in this study. More clinical studies are needed to confirm this part of clinical results in the future. However, exploring the risk factors of short-term readmission in patients with HF can provide some theoretical guidance for the prognosis of patients with HF in the future. We should pay close attention to high-risk groups.

The innovation of this study lies in the following aspects: firstly, the short-term readmission of HF patients was used as a dependent variable. Furthermore, this study was a longitudinal study that can identify risk factors for short-term readmission. Thirdly, this study used R software to simulate the RCS of the relationship of risk factors and short-term readmission. Fourthly, this study may provide some suggestions for the prognosis of Chinese HF patients. However, this study also has some limitations. The subjects of this study were from the same hospital, and thus, our results extrapolated to other populations should be treated with caution. There may be some factors that influence short-term readmission in patients with HF that have not been considered.

## Conclusion

Our study showed that NYHA class, diabetes, renal function indicators UA and Cr, RDW-SD, Na, and CCI were predictors of short-term readmission risk in patients with HF. At the same time, the nonlinear relationship between predictors and short-term readmission risk may provide the theoretical basis for the prognosis of patients with HF.

## Data availability statement

All data are provided by public database, the database is available at PhysioNet: https://doi.org/10.13026/8a9e-w734.

## Ethics statement

The studies involving human participants were reviewed and approved by Ethics Committee of the Fourth People’s Hospital of Zigong. Written informed consent for participation was not required for this study in accordance with the national legislation and the institutional requirements. Written informed consent was obtained from the individual(s) for the publication of any potentially identifiable images or data included in this article.

## Author contributions

XN: write a manuscript. HW, NS, RZ: Data curation. TW and YL: Edit and polish articles. All authors contributed to the article and approved the submitted version.

## Conflict of interest

The authors declare that the research was conducted in the absence of any commercial or financial relationships that could be construed as a potential conflict of interest.

## Publisher’s note

All claims expressed in this article are solely those of the authors and do not necessarily represent those of their affiliated organizations, or those of the publisher, the editors and the reviewers. Any product that may be evaluated in this article, or claim that may be made by its manufacturer, is not guaranteed or endorsed by the publisher.

## Abbreviations

COPD, Chronic obstructive pulmonary disease; CCI, Charlson comorbidity index; BMI, Body mass index; Cr, Creatinine; UA, Uric acid; GFR, Glomerular filtration rate; RDW-CV Coefficient of variation of red blood cell distribution width; RDW-SD Standard deviation of red blood cell distribution width; HGB, Hemoglobin; Ca, Calcium; K, Potassium; Na, Sodium; TG, Triglyceride; LDL-C, Low-density lipoprotein cholesterol; HDL-C, High-density lipoprotein cholesterol.
