# Supplementary material for: Exploring risk factors of short-term readmission in heart failure patients: A cohort study
Source: Front Endocrinol (Lausanne). 2022 Nov 28;13:1024759. doi: 10.3389/fendo.2022.1024759 (PMC9742544; doi:10.3389/fendo.2022.1024759)
Supplement: Supplementary file 1 [file DataSheet_1.pdf]

The difference of each index between the readmission group within 6 months and the non-readmission group within 6 months.

```
FREQUENCIES VARIABLES=BMI cr urea UA gfr RDWCV RDWSD HGB Ca K Na tg cholesterol ldlc hdlc
  /NTILES=4
  /STATISTICS=MEDIAN
  /ORDER=ANALYSIS.
```

频率

统计

		BMI	cr	urea	UA	gfr	RDWCV	RDWSD	HGB	Ca	K	Na	tg	cholesterol	ldlc	hdlc
个案数	有效	1065	1065	1065	1065	1065	1065	1065	1065	1065	1065	1065	1065	1065	1065	1065
	缺失	0	0	0	0	0	0	0	0	0	0	0	0	0	0	0
中位数		20.8889	83.1000	7.5700	444.0000	68.8400	14.4000	47.5000	118.0000	2.2800	3.8400	139.2000	.9700	3.6300	1.7800	1.0800
百分位数	25	18.6643	63.0500	5.6700	352.0000	44.5100	13.5000	44.9000	102.0000	2.1700	3.4900	136.4000	.7300	3.0300	1.3600	.8550
	50	20.8889	83.1000	7.5700	444.0000	68.8400	14.4000	47.5000	118.0000	2.2800	3.8400	139.2000	.9700	3.6300	1.7800	1.0800
	75	23.4566	115.6000	10.8600	557.0000	93.6200	15.5000	50.9000	133.0000	2.3900	4.2800	141.7000	1.2800	4.3650	2.3300	1.3100

频率表

BMI

频率

统计

		BMI	cr	urea	UA	gfr	RDWCV	RDWSD	HGB	Ca	K	Na	tg	cholesterol	ldlc	hdlc
个案数	有效	662	662	662	662	662	662	662	662	662	662	662	662	662	662	662
	缺失	0	0	0	0	0	0	0	0	0	0	0	0	0	0	0
中位数		20.3954	92.9000	8.6100	474.0000	58.0250	14.4000	48.1000	116.0000	2.3000	3.9000	138.8000	.9100	3.4600	1.6900	1.0600
百分位数	25	18.3655	69.0750	6.2400	374.0000	39.1975	13.7000	45.4750	100.0000	2.1900	3.5775	135.5000	.6900	2.8675	1.2600	.8600
	50	20.3954	92.9000	8.6100	474.0000	58.0250	14.4000	48.1000	116.0000	2.3000	3.9000	138.8000	.9100	3.4600	1.6900	1.0600
	75	23.4375	130.9250	12.3100	600.5000	83.0850	15.8000	52.1000	129.0000	2.4100	4.3700	141.0000	1.3100	4.2300	2.2000	1.3000

频率表

BMI

	BMI	cr	urea	UA	gfr	RDWCV	RDW
曼-惠特尼 U	334427.000	309290.000	306512.500	312759.500	306785.500	330461.000	323403
威尔科克森 W	553880.000	876935.000	874157.500	880404.500	526238.500	898106.000	891048
Z	-1.795	-4.290	-4.566	-3.946	-4.538	-2.189	-2.
渐近显著性（双尾）	.073	.000	.000	.000	.000	.029	[illegible]

a. 分组变量：分组

检验统计[a]

RDWSD	HGB	Ca	K	Na	tg	cholesterol	ldlc	hdlc
3403.500	325528.500	331905.000	326262.000	325332.500	335868.500	323653.000	324718.500	346428.500
1048.500	544981.500	899550.000	893907.000	544785.500	555321.500	543106.000	544171.500	565881.500
-2.889	-2.679	-2.046	-2.606	-2.698	-1.652	-2.864	-2.759	-.604
.004	.007	.041	.009	.007	.099	.004	.006	.546

The difference of each index between the readmission group within 6 months and the non-readmission group within 3 months.

频率

统计

		BMI	cr	urea	UA	gfr	RDWCV	RDWSD	HGB	Ca	K	Na	tg	cholesterol	ldlc	hdlc
个案数	有效	1309	1309	1309	1309	1309	1309	1309	1309	1309	1309	1309	1309	1309	1309	1309
	缺失	0	0	0	0	0	0	0	0	0	0	0	0	0	0	0
中位数		20.8209	84.6000	7.6200	442.0000	67.8800	14.3000	47.6000	118.0000	2.2900	3.8400	139.2000	.9600	3.6200	1.7700	1.0800
百分位数	25	18.6090	63.7500	5.7500	354.5000	43.9400	13.6000	45.0000	102.0000	2.1800	3.5000	136.4000	.7100	3.0200	1.3400	.8700
	50	20.8209	84.6000	7.6200	442.0000	67.8800	14.3000	47.6000	118.0000	2.2900	3.8400	139.2000	.9600	3.6200	1.7700	1.0800
	75	23.5556	116.6500	10.9750	556.5000	92.2400	15.5000	50.9000	132.0000	2.4000	4.2700	141.6500	1.2900	4.3300	2.3050	1.3200

频率表

BMI

	频率	百分比	有效百分比	累计百分比

频率

统计

		BMI	cr	urea	UA	gfr	RDWCV	RDWSD	HGB	Ca	K	Na	tg	cholesterol	ldlc	hdlc
个案数	有效	418	418	418	418	418	418	418	418	418	418	418	418	418	418	418
	缺失	0	0	0	0	0	0	0	0	0	0	0	0	0	0	0
中位数		20.2604	98.9000	9.1300	486.5000	55.9600	14.6000	48.4000	115.0000	2.2900	3.9200	138.3500	.9200	3.4350	1.6550	1.0600
百分位数	25	18.3412	70.4750	6.4050	384.0000	37.2750	13.8000	45.6000	99.0000	2.1900	3.6200	135.0000	.7000	2.8325	1.2500	.8400
	50	20.2604	98.9000	9.1300	486.5000	55.9600	14.6000	48.4000	115.0000	2.2900	3.9200	138.3500	.9200	3.4350	1.6550	1.0600
	75	22.8928	135.9000	13.1975	627.0000	81.0375	15.9000	52.8000	128.0000	2.3900	4.4100	141.0000	1.3325	4.1625	2.1825	1.2625

频率表

检验统计[a]

	BMI	cr	urea	UA	gfr	RDWCV	RDWSD	HGB	Ca	K
曼-惠特尼 U	254927.500	235819.000	228207.000	233317.500	233064.500	244446.500	243895.000	250472.000	270018.000	246747.5
威尔科克森 W	342498.500	1093214.000	1085602.000	1090712.500	320635.500	1101841.500	1101290.000	338043.000	1127413.000	1104142.5
Z	-2.102	-4.254	-5.112	-4.536	-4.564	-3.283	-3.344	-2.604	-.401	-3.0
渐近显著性（双尾）	.036	.000	.000	.000	.000	.001	.001	.009	.688	.0

a. 分组变量：分组

IBM SPSS Statistics 处理程序就绪 | 个案：100 | Unic

9:06

检验统计[a]

DWCV	RDWSD	HGB	Ca	K	Na	tg	cholesterol	ldlc	hdlc
4446.500	243895.000	250472.000	270018.000	246747.500	238343.500	268756.500	248131.500	250719.000	258676.500
1841.500	1101290.000	338043.000	1127413.000	1104142.500	325914.500	356327.500	335702.500	338290.000	346247.500
-3.283	-3.344	-2.604	-.401	-3.023	-3.970	-.544	-2.867	-2.576	-1.679
.001	.001	.009	.688	.003	.000	.587	.004	.010	.093

信息区域

S Statistics 处理程序就绪 | 个案：100 | Unicode:ON

9:06

中 星期五

2022-10-14
